# Vivisection Once More

**Published:** 1881-04

**Authors:** 


### Editorial.

---

### VIVISECTION ONCE MORE.

A DISTINGUISHED member of the State Senate has favored us with a copy of a lecture on this subject, by Mr. Henry Berg, which was " delivered in the Assembly Chamber of New York, at Albany, before a joint committee of both houses of the Legislature, February 19, 1880." The various phases of the question have been so ably and thoroughly considered by the Parliamentary committee in England, by Professor Dalton in this country, and by the many medical journals, both there and here, that almost nothing remains to be said concerning it. Mr. Berg, like most other fanatics, has undoubtedly done a deal of good in his own curious way. By calling attention to the cruel treatment of horses, to the brutal method of transporting live stock by rail, and to the wanton slaughter of birds, under the pretense of " sport," he has aroused public sentiment, and instituted reforms for which much praise is cheerfully accorded him. But as long as men are born with carniverous tastes, so long will the weaker animals be sacrificed to gratify their appetites. As long too, as humanity is afflicted with disease, so long will it eagerly grasp at every means of relief, no matter if that must be obtained at the cost of suffering.

It is quite possible that a few incompetent or careless students have been unnecessarily cruel in their methods of investigation, but this does not alter the fact that invaluable knowledge of drugs, of operative procedures, and of various means for the relief of suffering, has been gained by experiment upon animals. This is too well known to require any demonstration at present. Nor do we propose to reply to the incoherent tirade which Mr. Berg calls a " lecture." The few assertions which seem to be worthy of any attention have been refuted more than once, as already stated. But some of the arguments now advanced are so thoroughly, original, and yet so characteristic of this enthusiastic gentleman, that they cannot be dismissed without a word of comment.

There are a number of these which might be referred to, but the space in our pages and the time of the subscribers is too valuable to permit the mention of more than a single example. For the sake of fairness we give the writer's own words, exactly as they appear on page fifteen of the pamphlet:

"As another proof of the profane extremes to which these dissectors of living animals will go, Robert McDonald, M. D., on being questioned, declared that he had opened the veins of a *dying person*, remember, and had injected the blood of an animal into them many times, and had met with brilliant success. In other words, this potentate had discovered the means of thwarting the decrees of Providence, where a person was dying, and snatching away from its Maker a soul which He had called away from earth!"

This may be an excellent argument against vivisection, to those who have come to the conculsion that life is not worth living, but if any one of the "joint committee of both houses" were placed in a similar position and left to his own choice, we strongly suspect that in view of the uncertainty of his future destination, he would promptly request the nearest doctor to "snatch" him—and that too, without much delay.

There is, however, a danger that undue influence may be brought to bear upon our law-makers, by persuasive allurements which charm away real argument.

The officers and members of the various societies for the prevention of cruelty to animals, would smile approvingly upon the Senator or Representative, who caused the killing of an animal to become a capital crime. Now, when we remember that these societies are composed largely of energetic ladies and enticing maidens, what wonder is it, that the heart of the modern Lycurgus should soften in sympathy for "our dumb friends," but especially in a tender regard for other friends who are very far from dumb? A man may indeed be strong in his own convictions, he may be high above the ordinary forms of bribery, but who can resist the eloquent plea of a lady acquaintance in behalf of her pet pug? It is against the seductive influence thus exerted by the members of these societies, that, in a modest way, we would presume to offer a word of warning.

